# Selective sweeps for mutations increasing height impede identification of causative mutations for fertility and other correlated traits in cattle

**DOI:** 10.1186/s12711-025-01004-x

**Published:** 2025-10-07

**Authors:** Mehrnush Forutan, Elizabeth M. Ross, Amanda J. Chamberlain, Geoffry Fordyce, Bailey N. Engle, Loan T. Nguyen, Ben J. Hayes

**Affiliations:** 1 Queensland Alliance for Agriculture and Food Innovation, The University of Queensland, St Lucia, QLD Australia; 2 Centre for AgriBiosciences, Agriculture Victoria, Bundoora, VIC Australia; 3 School of Applied Systems Biology, La Trobe University, Bundoora, VIC 3083 Australia; 4 USDA, ARS, U.S. Meat Animal Research Center, Clay Center, NE 68933 USA

## Abstract

**Background:**

Fertility, growth and body composition are key drivers of profitability in beef cattle. With the aim of identifying causative mutations underpinning variation in these traits, we integrated multi-trait genome-wide association analysis (M-GWAS) in a cohort of 28,351 multibreed beef cattle with imputed whole genome sequence (WGS) data, with expression quantitative trait loci (eQTL) summary statistics from 489 indicine cattle using the same WGS variants. An additional aim was to provide insights into the biological basis for the association between growth, metabolism, and reproductive development. First, we conducted M-GWAS for live weight, hip height, body condition score and heifer puberty at approximately 600 days. Subsequently, focusing on a 2 Mb region around the lead GWAS SNP we identified the top eQTL in each region. Through iterative conditional analysis, we successively integrated these variants into individual single trait GWAS and further analysed expression and trait information using conditional and joint GWAS analysis. This iterative process continued until no additional significant SNPs emerged from the M-GWAS.

**Results:**

Fifteen candidate genes were identified, including *IRAK3*, *HELB*, *HMGA2*, *LAP3, FAM184B, LCORL*, *PPM1K*, *ABCG2*, *MED28*, *PLAG1*, *BPNT2*, *UBXN2B, CTNNA2*, *SNRPN*, and *SNURF*. When we investigated the number of eQTL in blood associated with these genes, *IRAK3*, *HELB*, *PPM1K*, *ABCG2*, *MED28*, *BPNT2*, and *UBXN2B* were associated with a single eQTL, while *ABCG2* was clearly associated with two eQTLs (Bonferroni corrected *P* < 1 × 10^–10^). However, the identification of potential QTLs in these regions was impeded by extensive localised linkage disequilibrium. Analysis of extended haplotype homozygosity in the regions revealed this extended linkage disequilibrium was likely the result of recent strong selection, in most cases for the allele increasing height (Chi-square *P* = 0.000967).

**Conclusions:**

This observation sheds some light on why it has been so difficult to identify mutations affecting fertility, and other traits that are pleiotropic with height, in cattle.

**Supplementary Information:**

The online version contains supplementary material available at 10.1186/s12711-025-01004-x.

## Background

Fertility, growth and body composition are key drivers of production in beef cattle. Several genome-wide association studies (GWAS) and quantitative trait loci (QTL) mapping studies in cattle, and other mammals have reported numerous genomic regions associated with these traits [1–3]. However, in many cases the causal gene is unclear and often the related physiological pathway is unclear. For instance, several studies have reported genetic variants near the gene *LCORL*, implicating its effect on size in several species [4, 5], but the mechanism by which they do this is unknown [5]. Linking the effect of SNPs to gene function is not straightforward without additional data, especially, as many of these trait-associated variants fall into non-coding regions of the genome with no direct influence on protein structure or function.

Increasing the sample size, using imputed whole genome sequence (WGS) data, and performing multi-trait GWAS analysis, could result in greater power to detect QTL, more precise mapping of associated genomic regions, and additional information to prioritize genes associated with traits [2, 5, 6]. While direct utilization of WGS data is feasible, imputation is particularly advantageous in large-scale and cost-effective studies where sequencing all samples is impractical.

Trait-associated SNPs are frequently expression quantitative trait loci (eQTLs) [7–10], suggesting a functional basis for their association with traits, and that incorporating expression information may help identify causative mutations. Variants located outside of protein-coding regions can influence gene expression by affecting regulatory elements such as enhancers, promoters, and transcription factor binding sites. These variants can also alter the accessibility of DNA to transcription factors, modify chromatin structure, or impact RNA processing, ultimately influencing gene activity [11].

Identification of causative mutations affecting performance traits in single breed cattle populations has been impeded by extensive linkage disequilibrium (LD) resulting from recent contractions in effective population size [12]. In particular, taurine breeds exhibit higher LD (r^2^ = 0.45) at short distances between markers (< 10 kb), compared to indicine (r^2^ = 0.25) and composite breeds (r^2^ = 0.32), based on high-density SNP genotypes [13]. *Bos indicus* and *Bos taurus* crossbreds offer a promising alternative for the discovery of causal variants, as they tend to exhibit lower LD particularly relative to pure bred *Bos taurus* populations such as Holstein and Angus [12]. The reduced LD should enable more precise QTL mapping, perhaps enhancing our ability to pinpoint mutations influencing the traits.

Here, using 28,351 well-phenotyped *Bos indicus* (indicine), *Bos taurus* (taurine) and crossbred cattle, with imputed WGS data, we set out to map pleiotropic loci associated with body condition score (BCS), weight, hip height, and heifer puberty. We then aimed to integrate the results with publicly available eQTL summary statistic data [9], to identify significant SNPs and prioritized genes likely to mediate pleiotropic effects on these traits.

## Methods

### Phenotypic data

For this study, heifers grazing rangelands in commercial business across northern Australia, including across all of Queensland, parts of the Northern Territory, and the Pilbara region of Western Australia were assessed for fertility, weight, height, and BCS [14]. The 28,351 heifers enrolled included crossbred and 8046 purebred heifers from at least 14 breeds, i.e. Angus, Belmont Red, Brahman, Charolais, Droughtmaster, Hereford, Limousin, MurrayGrey, SantaGertrudis, Shorthorn, Wagyu, Boran, Senepol, and Tuli (all breeds with variable genetic distances and diversity parameters [12]). Traits measured from 2015 to 2020 on the heifers included live weight (kg), hip height (mm) and body condition score (BCS, 1–5 scale) at an average of 600 days, and a heifer puberty trait (Table 1). The latter was cycling or not cycling by an average of 600 days assessed by the presence or absence of a pregnancy or a *corpus luteum in non-pregnant heifers* using ultrasound scanning with a 10 Mz, 60 mm, rectal probe (Honda 2200V) [15]. To maximise genetic variation, the trait was measured as near as possible when logistically feasible when an estimated 50% of the heifers were pubertal (targeting an average group live weight of ~ 320kg), i.e., at 1 to 2.5 years of age. Further details on the phenotypes are described in Copley et al. [14].

**Table 1:** The summary statistics for phenotypes

Trait	Number of records	Average	Minimum	Maximum	Standard deviation
Body condition score (BCS)	24,602	3	0.5	5	0.47
Height	24,325	1294.07	950	1850	61.28
Weight	24,593	306.81	135	730	48.85
Heifer puberty	28,078	0.42	0	1	0.49

### Genotyping

All heifers were genotyped with the 35 k or 50 k Trop-Beef SNP array by Neogen, Australasia. SNPs were removed if more than 10% of the genotypes were missing for that SNP. If an individual genotype had a GenCall (GC) score lower than 0.6, it was set to missing and recovered by imputation. Genotypes were imputed up to 709,768 SNPs (bovine high-density (HD) array) using the findhap [16] software and a panel of 4506 cattle from relevant breeds that were genotyped with the Bovine HD array. This panel of HD SNPs was obtained by removing the SNPs that had less than 10 copies of the minor allele in the imputation reference panel, and the SNPs that had more than 10% missing genotypes. All breeds in the heifer population were represented by at least 50 animals in the Bovine HD imputation reference set. The accuracy of imputation was at least 93% for all breeds/crossbreds, assessed by cross validation. All genotypes were then imputed to WGS variants using the 1000 Bull Genomes Run8, TaurIndicus reference set [17], with 600 Holstein and 400 Simmental animals removed to avoid over-representation of these genomes in the imputation, such that 1261 whole genome sequenced animals remained. Eagle [18] was used for phasing and Minimac3 [19] for imputation. Sequence variants with fewer than 4 copies of the minor allele were removed prior to imputation in an attempt to avoid including sequencing errors in the data set. Overall, 49.8 million variants were imputed. We conducted GWAS using all imputed variants to maximize the potential for discovery and to avoid prematurely excluding variants that might carry biologically relevant signals. To ensure the robustness of our findings, we interpreted results and drew conclusions only for variants with high imputation quality. Only variants with minor allele frequency (MAF) > 0.0005 (44.7 million) were used for GWAS study.

### Genome-wide association analysis

For each trait, a linear mixed model was performed using *mlma* package in GCTA [20], fitting each sequence variant as a covariate, one at a time, and testing for association with each trait as follows:

1 $$\mathbf{y}={\mathbf{1}}_{\mathbf{n}}\upmu +\mathbf{X}{\varvec{\upbeta}}+\mathbf{Z}\mathbf{g}+{ \mathbf{W}}_{\mathbf{i}}{{\varvec{\upalpha}}}_{\mathbf{i}}+\mathbf{e},$$

In Eq. (1), **y** is the vector of phenotypic values of the animals, ${\mathbf{1}}_{{\varvec{n}}}$ is an n × 1 vector of 1s (n = number of animals with phenotypes), μ is the overall mean, **X** is an n × x matrix of fixed covariates, **β** is a length x vector of fixed effects, **Z** is a design matrix for the random additive genetic effects, and **g** is a vector of random additive genetic effects assumed to be distributed as ∼*N*(0, **G** ${\sigma }_{g}^{2}$), where **G** is the genomic relationship matrix (**GRM**) among animals calculated using the GCTA program for imputed HD SNP data, fitted to account for population structure (where each element of **G** is the proportion of genome shared by each pair of individuals and ${\sigma }_{g}^{2}$ is the genetic variance). ${\mathbf{W}}_{\mathbf{i}}$ is a vector of genotypes for each animal at the ith variant, ${{\varvec{\upalpha}}}_{\mathbf{i}}$ is the coresponding additive effect (fixed effect) of the variant. The genotypes at each locus were coded as 0, 1, or 2, representing the number of copies of a particular allele carried by an individual. **e** is a random vector of length n as ∼ *N*(0, ${\upsigma }_{\text{e}}^{2}$ **I**), where ${\upsigma }_{\text{e}}^{2}$ represents non-genetic variance due to non-genetic effects assumed to be acting independently on animals. For all four traits, contemporary group, heterozygosity and the first four principal components (PCs) of genotypes, were considered as fixed covariates. The first four PCs were included to account for the effect of breed divergence (separation between taurine and indicine), within breed or sub-populations divergence, and finer population differences such as family structure, or even recent selection, and management group [21]. The contemporary group was defined as the herd-year effects. The heterozygosity for each animal was calculated as the proportion of heterozygotes loci across the genome, accounting for hybrid effects.

### Multi trait analysis

We performed a multi-trait analysis (M-GWAS) according to a previously described approach [2] using WGS variant effects estimated from the four single-trait GWAS to identify pleiotropic variants that affected the traits studied. The M-GWAS X^2^ statistic with 4 degrees of freedom (equal to the number of traits analysed) was calculated as: X^2^ = ${{\varvec{t}}}_{{\varvec{i}}}^{{\varvec{T}}}$ **V**^−1^**t**_i_, where ${{\varvec{t}}}_{{\varvec{i}}}^{{\varvec{T}}}$ is a transpose vector of${{\varvec{t}}}_{{\varvec{i}}}$, the signed t-values of the effects of the ith sequence variants for the 4 traits and **V**^**−1**^ is the inverse of the 4 × 4 correlation matrix w here the correlation was calculated over all estimated sequence variant effects (signed t-values) between each pair of traits. In this approach, the X^2^ statistics are calculated based on SNP effect estimates rather than requiring each individual to have phenotypic records for all traits. Instead, it requires each marker to have effect estimates across all traits.

### Evidence for expression and trait pleiotropy

We used expression quantitative trait loci (eQTL) summary statistics from a previous study [9] where gene expression levels in whole blood samples had been profiled for 489 indicine heifers and cows using RNA-seq. Only cis eQTLs—variants located within a 2 Mb upstream and downstream window around the gene start site—were included, and association mapping was performed using the same whole-genome sequence (WGS) variants as used in the M-GWAS described above. We assumed that if a significant SNP in a region influences the phenotype through gene expression (i.e., it is an eQTL) and is perfectly tagged by a GWAS QTL, then including this variant in the model should eliminate the association of nearby SNPs with the phenotype—meaning that these neighboring SNPs would no longer show significant effects when fitted simultaneously. To test this hypothesis, we considered three major association peaks located on Chr 5, 6, and 14. Within 2Mb regions centred around the most significant variants identified from the M-GWAS, we preselected variants showing strong association signals (p < 1 × 10⁻⁹; SNP set1). We then searched for the most significant eQTLs previously reported for these regions [9], and included them in a list of putative causal variants (SNP set2). Conditional and joint association (cojo) analysis using single trait GWAS summary statistics was then performed for all traits to assess the association of SNPs conditional on those in SNP set2. Subsequently, the M-GWAS was re-calculated to test the effects of the remaining variants across traits after fitting the identified SNPs (SNP Set2). During each cojo step, variants in LD with SNPs from the previously identified list (SNP set2) were excluded from the single-trait GWAS results and, consequently, from the M-GWAS. As a result, in subsequent rounds of M-GWAS, different candidate variants could reach significance. This iterative refinement continued until no further QTLs were identified at the defined threshold (*P* < 1 × 10^–9^, accounting for multiple test correction, FDR = 0.05, ~ 44 million SNPs).

### Pairwise correlations between SNP effect estimates across traits

To investigate the similarity of SNP effects between traits, we estimated pairwise correlations using the genome-wide signed SNP t-values (SNP effect estimates divided by the standard error of estimates) obtained from single-trait GWAS for BCS, heifer puberty, height, and weight. T-values were aligned across traits, and similarity of SNP effects were calculated as the Pearson correlation coefficients between corresponding SNP t-values for each trait pair. Genome-wide similarity were assessed using the full set of imputed WGS variants across all traits. Additionally, to explore the heterogeneity of correlations across the genome, we calculated correlations separately for each autosome. The correlations were also calculated for the subset of lead QTLs identified through the integration of GWAS and eQTL analyses. All correlation computations were conducted in R, using the cor () function, and results were visualized to highlight regions with strong or divergent correlations between traits.

### Conditional analysis of eQTL summary statistics results

Conditional analysis of eQTL summary statistics involved using the *cojo-cond* option in the GCTA program to refine genetic associations with specific genes. In this analysis, SNPs of interest, identified through the integration of eQTL and M-GWAS from the previous analysis, were incorporated into conditional models for their corresponding genes. This approach enables the examination of whether additional genetic variants contribute to gene expression variation beyond the primary eQTL signal.

### Extended haplotype homozygosity decay

We extracted the phased, imputed WGS data for the 2 Mb regions surrounding the lead QTLs—identified through the integration of GWAS and eQTL analyses—on chromosomes 5, 6, and 14. To measure how quickly haplotypes decay as we move away from the core SNP position, we calculated extended haplotype homozygosity (EHH) [22] for each QTL allele using the *calc_ehh* function of rehh package in R [23]. The differences in EHH between alleles at the core SNP was calculated by determining the area under the curve (AUC), using the *trapz* function from the pracma library in R [24]. 

## Results

### Genome-wide association studies

There were 114, 6405, 1683, and 4142 significant SNP for BCS, height, weight, and heifer puberty, respectively (*P* < 1 × 10^–9^, corresponding to an FDR threshold of 0.05 based on Bonferroni correction for ~ 44 million SNPs) [See Additional file 1, Table S1 and Additional file 2, Figure S1].

**Fig. 1:**
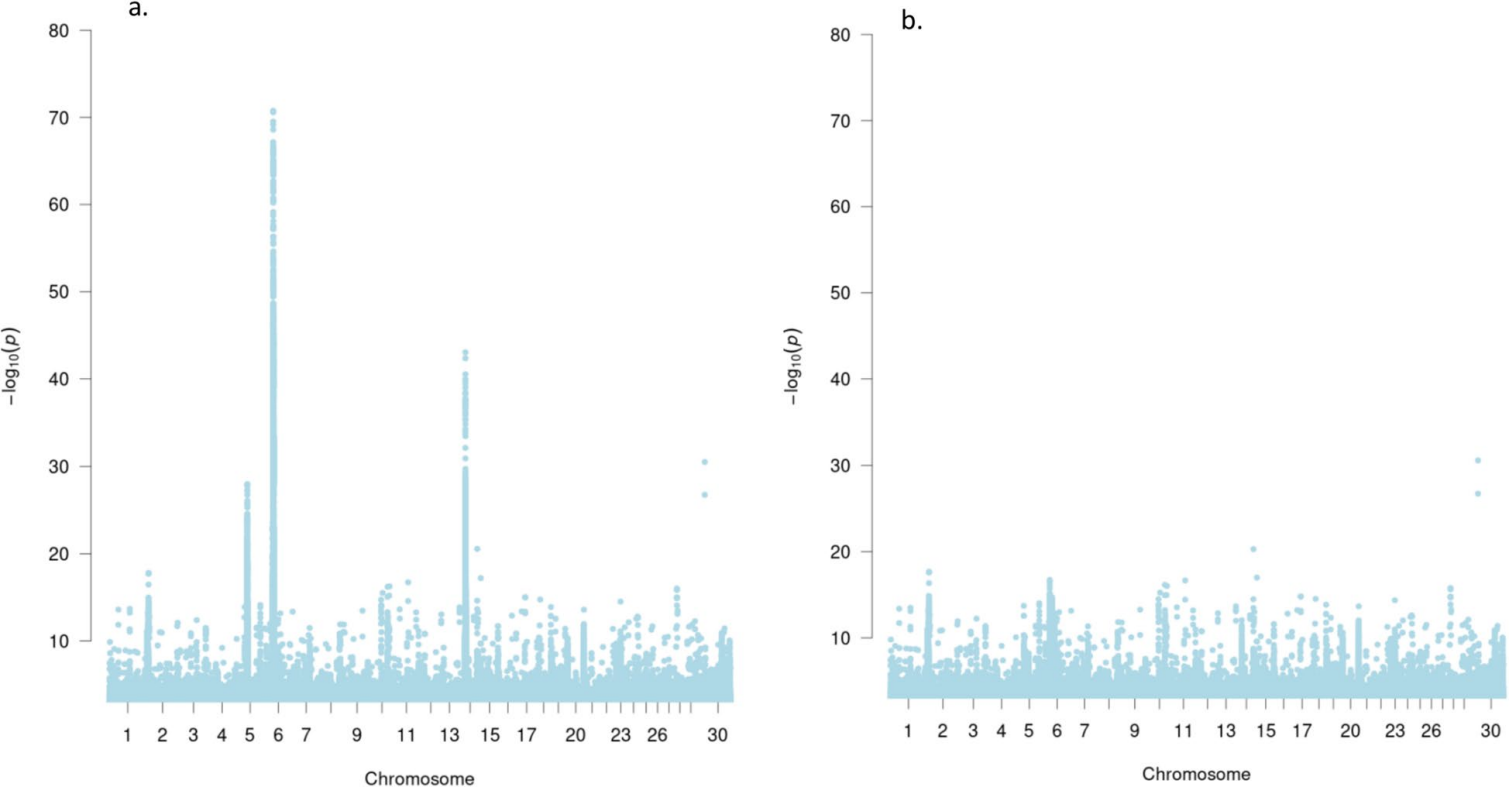
Manhattan plots depicting the Multi-trait GWAS analysis for height, weight, body condition score (BCS), and heifer puberty in 28,351 multibreed cattle. Before (**a**) and after conducting conditional analysis (**b**)

The major genomic region associated with BCS was located on Chr 5, spanning approximately 47.4 to 47.7 Mb. with the top SNP located at Chr5:47,613,200. For weight, significant association peaks were identified on Chr 6 (33.8–39.5 Mb) and Chr 30 (102.1–102.9 Mb), with top SNPs at Chr6:37,266,497 and Chr30:102,729,938, respectively. Height showed key signals on Chr 5 (47.4–49.2 Mb; top SNP: Chr5:47,841,198), Chr 6 (33–41 Mb, top SNP Chr6:37,204,143), and Chr 14 (22.7–24.98 Mb; top SNP: Chr14:233,895,88). Heifer puberty was associated with regions on Chr 5 (47.3–47.8 Mb; top SNP:47,845,759), Chr 6 (35.9–39.5 Mb; top SNP:37,998,696), Chr 14 (22.7–23.9 Mb; top SNP:23,300,304), and Chr 21 (0.7–2.2 Mb; top SNP:1,970,429) [See Additional file 1, Table S1 and Additional file 2, Figure S1]. While single-trait GWAS identified QTLs within broader chromosomal regions across the four traits, the multi-trait (M-GWAS) analysis was able to pinpoint more precise locations within these regions that are strongly associated with the four traits in question (Fig. 1a and Additional file 2, Figure S2). M-GWAS identified the most significant SNP on Chr 5 (*P* = 1.08 × 10^−28^), Chr 6 (*P* = 1.71 × 10^−71^) and Chr 14 (*P* = 8.78 × 10^−44^) at 47,845 kb, 37,266 kb, 23,338 kb, respectively [See Additional file 3, Table S2].

**Fig. 2:**
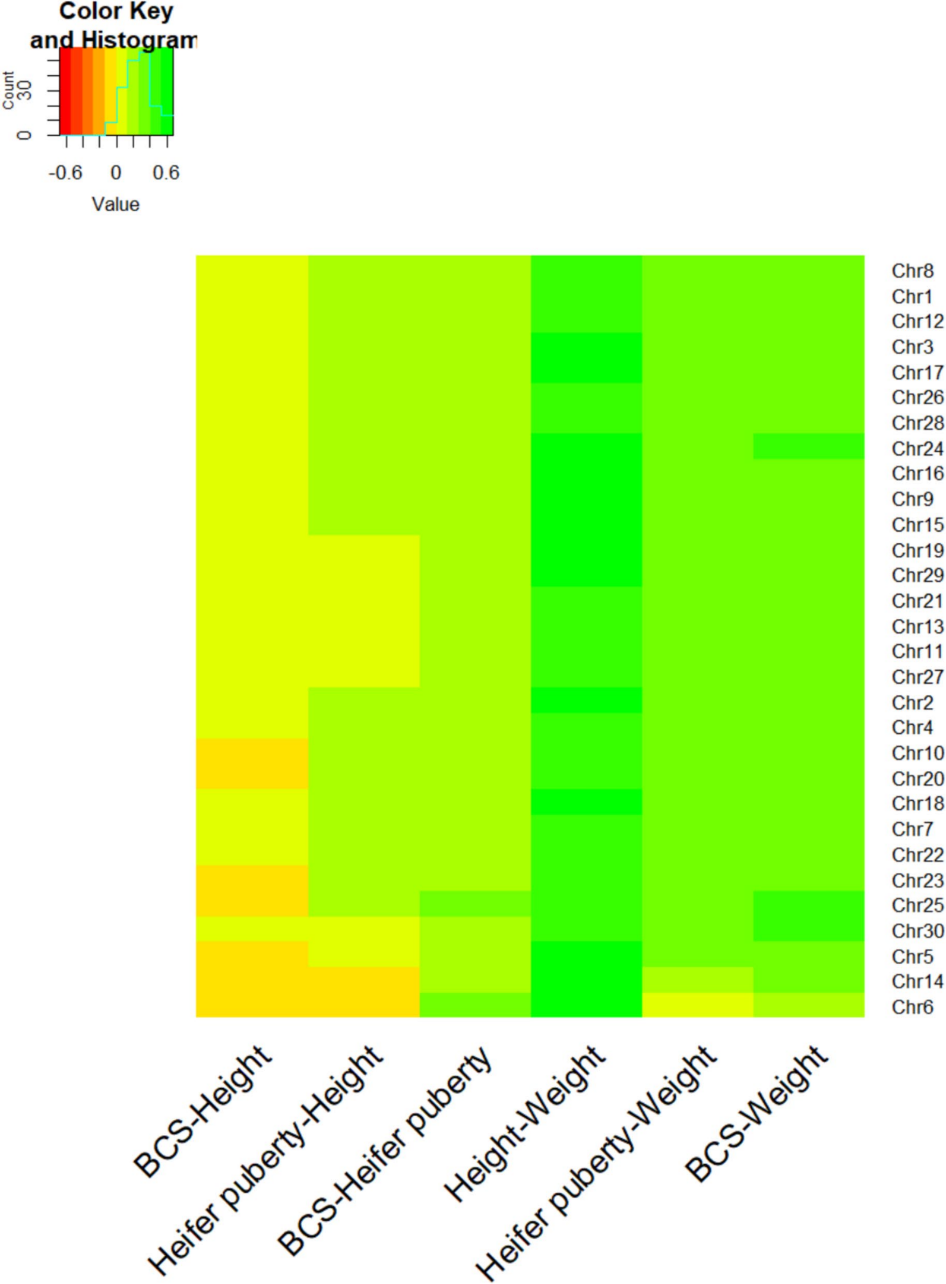
Correlation coefficients of SNP effects obtained from single-trait GWAS analysis of four traits (height, weight, body condition score (BCS), and heifer puberty) across chromosomes, illustrating patterns of positive and negative correlations among traits. The colour gradient applied in this graph effectively represents the range of correlation values, with red indicating lower correlations and green indicating higher correlations

The similarity of SNP effects between traits—obtained from single-trait GWAS analysis for BCS, heifer puberty, height, and weight—exhibit varying relationships both overall and across chromosomes. There was a low correlation between height and BCS (0.0134), height and heifer puberty (0.126), heifer puberty and BCS (0.207), moderate positive correlations between BCS and weight (0.361), heifer puberty and weight (0.311), and a strong positive correlation between height and weight (0.541). However, correlation between traits revealed a nuanced picture across chromosomes (Fig. 2). Positive correlations ranging from approximately 0.1 to 0.5 was observed across most chromosomes for the trait pairs, affirming the general trend of positive associations observed in the overall analysis. Despite this, specific chromosomes displayed notable negative correlations. For instance, BCS and height exhibited negative correlations up to -0.1 on chromosomes 5, 6, 10, 14, 20, 23, and 25. Similarly, negative correlations between heifer puberty and height, ranging from − 0.05 to − 0.08, were found on chromosomes 6 and 14.

### Evidence for expression and trait pleiotropy

We identified six lead candidate QTLs through an overlap with eQTL summary statistics which include one at 47.59 Mb on Chr 5, four at 37.11 Mb, 37.16 Mb, 37.69 Mb and 37.90 Mb on Chr 6 and one at 23.89 Mb on Chr 14, alongside three additional QTLs that emerged as top GWAS peaks from the M-GWAS analysis, located on Chr 5 (47.84 Mb), Chr 6 (37.26 Mb), and Chr 14 (23.33 Mb) [See Additional file 3, Table S3]. In the original M-GWAS, many SNPs on Chr 5 (47–49 Mb), Chr 6 (36–38 Mb), Chr 14 (22–24 Mb) were significant. However, after fitting the 6 lead QTL in these regions, no more significant SNPs remained (Fig. 1b). The correlation between each of the three top M-GWAS SNPs and each of the six lead QTLs, based on their effect sizes derived from single-trait GWAS analysis across four traits, revealed that all six lead QTLs were highly correlated with the top QTLs identified from M-GWAS [See Additional file 3, Table S4]. The apparent effects of the 9 QTLs on the 4 traits, as estimated in the original single-trait GWAS, are given in Additional file 3, Table S5. There were strong negative/positive correlations between traits for the 9 QTLs on chromosomes 5, 6, and 14, ranging from 0.90 to 0.98 [See Additional file 3, Table S6].

On Chr 5, the lead eQTL discovered through integrating GWAS and eQTL data was in an intron of Interleukin-1 receptor-associated kinase-3 (*IRAK3)* at position 47,593,069 bp, with significant effects [9] on gene expression of DNA helicase B (*HELB*) and *IRAK3* (with gene start sites of 47,519,482 bp and 47,624,668 bp, respectively). However, the most significant multi-trait GWAS peak on Chr 5 was located in gene the high mobility group AT-hook 2 gene (*HMGA2*) at position 47,845,893 bp, with no significant association found with any genes investigated in our eQTL study [9]. Additionally, in line with previous findings in indicine cattle [25] and pigs [26], *HMGA2* was either not expressed or expressed at significantly low levels in adult blood samples [9], so we could not find an eQTL for this gene in the current study. Although Brahman cattle (*Bos indicus* content > 0.80) and admixed or tropical composite animals displayed different combinations of genotypes at the two loci in *IRAK3* and *HMGA2* a Chi-square test examining the hypothesis of independent inheritance of these loci implied that they tend to be inherited together more often than expected by chance (df = 4, *P* < 2.2 × 10^−16^).

On Chr 6, one of the four lead eQTL discovered by integrating GWAS and eQTL data was located at 37,167,006 bp, 815 bp upstream of gene Leucine Aminopeptidase 3 (*LAP3*), with a significant effect on gene expression of Mediator Complex Subunit 28 (*MED28*) [9]. The second variant (Chr 6:37,115,973 bp) was located 1593 bp downstream of *LOC132345489* gene and was significantly associated [9] with expression of Protein Phosphatase, Mg2 + /Mn2 + Dependent 1K (*PPM1K)* and ATP Binding Cassette Subfamily G Member 2 (*ABCG2)* genes. The third variant was located close (133 kb) to Ligand Dependent Nuclear Receptor Corepressor Like (*LCORL*) gene, with significant effect on gene expression of *PPM1K* and *MED28* [9]. The fourth variant was located 122 kb away from *LOC132345490* gene, and had significant association with *ABCG2* gene expression [9]. However, the most significant GWAS peak on Chr 6 was located at position 37,266,497 bp within the Family With Sequence Similarity 184 Member B (*FAM184B*) gene*,* with no significant association found with any genes investigated in our eQTL study [9] [See Additional file 3, Table S3].

On Chr 14, the lead eQTL discovered by integrating GWAS and eQTL data was located at 23,897,198 bp, 14 kb upstream of gene 5'-Bisphosphate Nucleotidase 2 (*BPNT2)*, with a significant effect on *BPNT2* and UBX Domain Protein 2B* (UBXN2B)* [9] gene expression. Interestingly the most significant GWAS SNP was located in the Pleomorphic adenoma gene 1* (PLAG1)* gene, with a reported effect [9] on *UBXN2B* gene expression.

### Refining QTL discovery by incorporating selection signature profiles

**Fig. 3:**
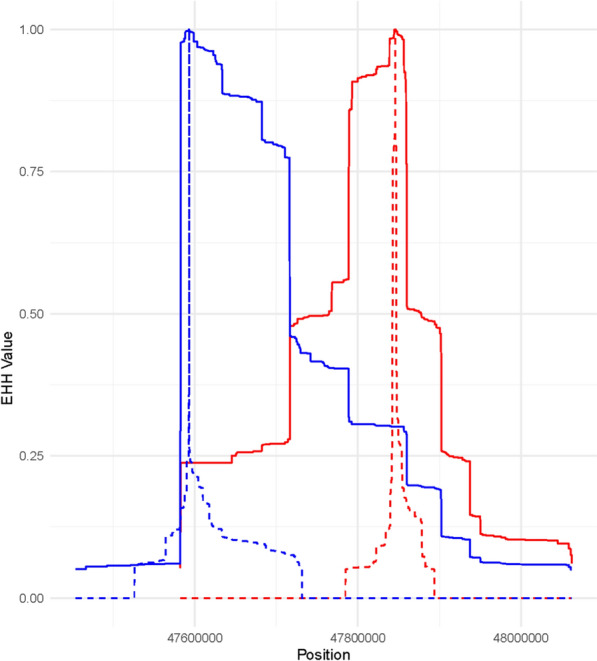
Extended haplotype homozygosity (EHH) decay for IRAK3 (blue) and HMGA2 (red) Loci. The solid line represents height-increasing allele vs. the dashed line shows the non-increasing-height allele

**Fig. 4:**
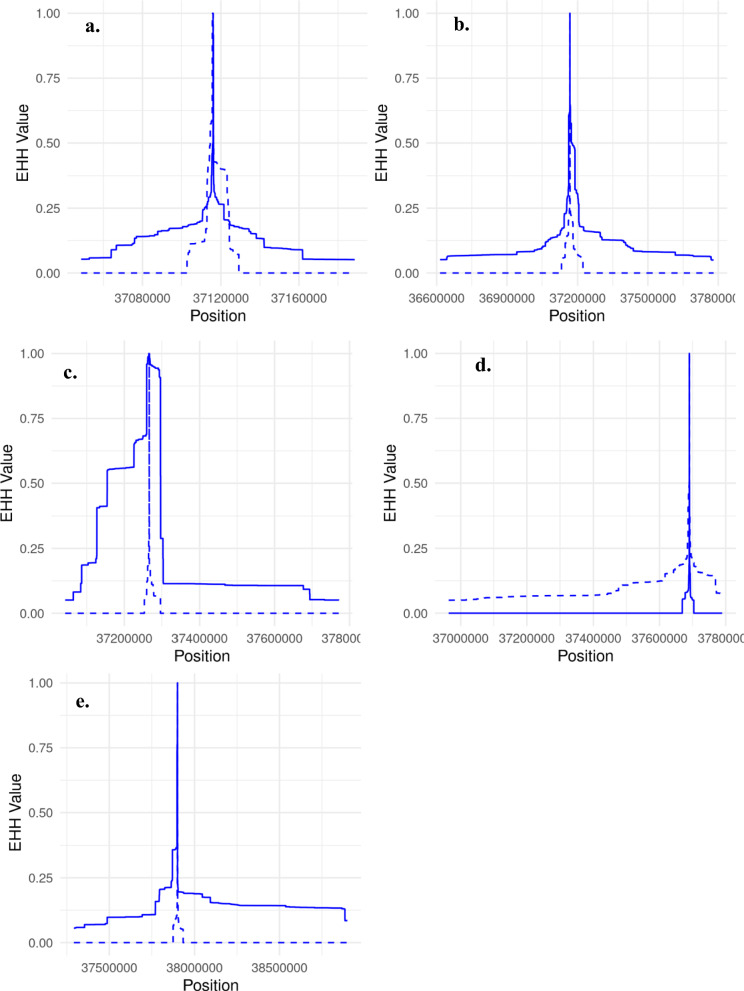
Extended haplotype homozygosity (EHH) decay for 5 candidates QTLs on Chr6 Candidates are located at (**a**) 37,115,973 bp, (**b**) 37,167,006 bp, (**c**) 37,266,497 bp, (**d**) 37,690,283 bp, and (**e**) 37,901,756 bp. The solid line represent height increasing allele vs. the dashed line shows the non-increasing height allele

**Fig. 5:**
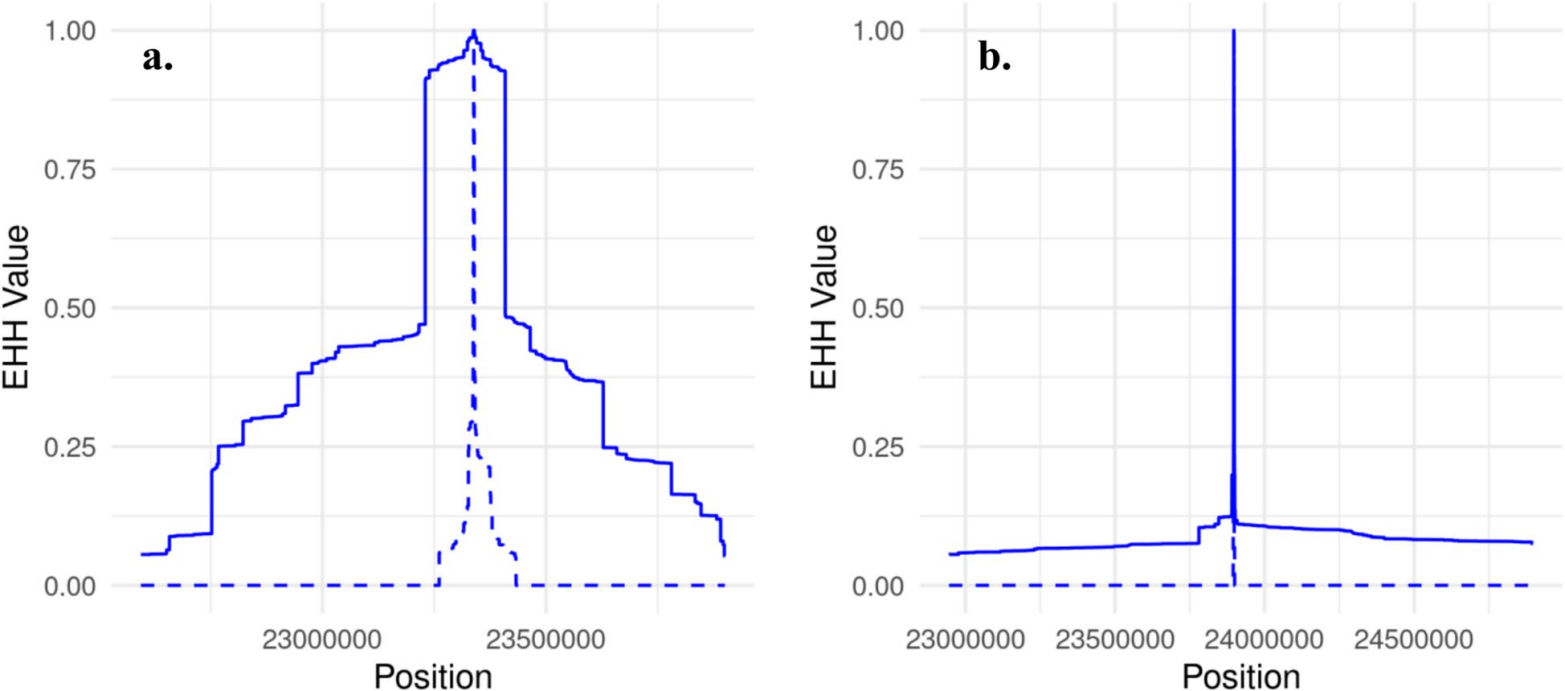
Extended haplotype homozygosity (EHH) decay on chromosome 14. For (**a**) PLAG1, and (**b**) BPNT2 loci. The solid line represent height increasing allele vs. the dashed line shows the non-increasing height allele

Sabeti et al. [22] suggested that quantifying the strength of selection signatures using extended haplotype homozygosity may assist in the identification of causative mutations underpinning trait variation. Regions under recent positive selection will show extended regions of high homozygosity because the advantageous allele rapidly increased in frequency, carrying along neighbouring alleles due to linkage disequilibrium. The extended haplotype homozygosity (EHH) decay profiles for the candidate QTLs on Chromosomes 5, 6, and 14 are shown in Figs. 3, 4, and 5, respectively. These profiles illustrate the rate at which haplotype homozygosity decreases around each candidate QTL allele in response to selection (or possibly drift), revealing distinct patterns that suggest varying levels of selective pressure and haplotype diversity. Notably, although the (height increasing) C allele at *IRAK3* SNP tended to show higher Area Under the Curve (AUC) values compared to (height increasing) T allele at *HMGA2* SNP (198,536.1 vs. 180,093.1), this difference was not statistically significant based on permutation tests (n = 10,000). On Chr 6, the difference in the EHH profile around the candidate pleiotropic QTLs reflects longer regions of extended homozygosity around the position Chr 6:37,266,497, where EHH decay extends over a larger region compared to other candidates on Chr6 (Fig. 4).

The EHH values for the height-increasing alleles were significantly higher across the regions of interest, suggesting longer regions of homozygosity (Wilcoxon signed rank *P* < 2.2 × 10^−16^). Out of the nine candidate QTLs, eight significantly exhibited longer extended regions associated with increased height (Chi-square *P* = 0.000967).

### Unravelling the genetic architecture of gene expression

To enhance our understanding of the genetic architecture underlying the expression of the genes *IRAK3*, *HELB*, *PPM1K*, *ABCG2*, *MED28*, *BPNT2*, and *UBXN2B*, we conducted a conditional eQTL analysis. In this analysis, we included the candidate lead QTLs identified through the overlap of eQTL summary statistics and GWAS peaks from the M-GWAS as covariates for each gene. By controlling for the effects of these lead eQTLs, our approach allowed us to assess the remaining genetic variation influencing gene expression, potentially uncovering secondary or novel eQTLs. Among them, the *ABCG2* gene stands out as it is influenced by two distinct eQTLs (Chr 6:37,115,973, Chr 6:36,400,549), whereas the other genes each have only one eQTL (Bonferroni corrected *P* < 1 × 10^–10^; [See Additional file 3, Table S3]).

### Correlations between SNP effects on gene expression and phenotypes

We calculated the correlation between the effects of six lead QTLs on expression of the corresponding gene (including *IRAK3*, *HELB*, *PPM1K*, *ABCG2*, *MED28*, *BPNT2*, and *UBXN2B)* and their effects on the phenotypic trait [See Additional file 1, Table S2]. Specifically, the correlation between the effects of candidate QTLs on height and expression of these genes showed a moderate positive correlation (0.45), suggesting that higher effects on gene expression are moderately linked to greater QTL effects on height. Conversely, a strong negative correlation (− 0.88) was observed with BCS, suggesting that QTLs significantly increasing gene expression are strongly associated with decreased BCS. In contrast, there was a negligible correlation (− 0.06) with heifer puberty, and a weak positive correlation (0.27) with weight.

## Discussion

In line with previous studies [2, 5, 9], our multi-trait GWAS analysis using sequence data demonstrated high power and reasonable resolution in identifying potential pleiotropic SNPs and genes affecting the traits under study.

On Chr 5, we identified two significant loci in the *HMGA2* and *IRAK3* genes. Both variants are linked to an increased likelihood of earlier puberty and are associated with lower weight and height but higher BCS. Our results indicate that these variants tend to be co-inherited more frequently than expected by chance (df = 4, Chi-square *P* < 2.2 × 10^−16^). We confirmed this finding by demonstrating that when SNP Chr 5:47,593,069 (*IRAK3* SNP) was included in the statistical model the other variant (Chr 5:47,845,893) had a non-significant effect (*P* < 0.003 in individual single trait GWAS). Given that these variants are in regions under selection [27], they might show patterns of co-segregation due to selective sweeps in the population, due to the selection for beneficial mutations in this region. The variants are within a previously reported 430 kb selective sweep (47,438,392–47,865,772 bp) spanning several genes including *HELB*, *IRAK3*, *TMBIM4*, *GRIP1*, and part of *HMGA2* [27]. Those authors suggested that the region, especially the *HELB* gene, has an impact in adaptation of tropical cattle to harsh environments such as high temperatures and high levels of ultraviolet (UV) intensity. Variants in the *HELB* gene have also been reported to be associated with fertility [9] and yearling weight [28] in *Bos indicus* cattle. The *HELB* gene encodes a DNA-dependent ATPase which catalyses the unwinding of DNA necessary for DNA replication, repair, recombination, and transcription [29].

Many authors (e.g. [30, 31]) have suggested that the origin of selection signatures will often be QTL for livestock and plant traits, potentially making them useful for identifying mutations affecting quantitative traits (though it should be noted Kemper et al. [32] did not find results supporting this hypothesis). We investigated extended haplotype homozygosity [22] surrounding the *IRAK3* and *HMGA2* QTL alleles, to investigate if the EHH results supported one gene over the other. The hypothesis was that the favourable allele (e.g. increasing height) will show longer regions of extended homozygosity due to recent positive selection, which has not been broken down by recombination as much as the allele associated with decreased height. Consequently, the AUC when comparing two variants could be higher for the allele that has more recently experienced strong selection. The AUC for the (height increasing) C allele at the *IRAK3* locus was slightly higher than that for the (height increasing) T allele at *HMGA2* locus. This indicates that the EHH decay around the C allele at the *IRAK3* locus extends over a larger region compared to the T allele at the *HMGA2* locus, suggesting a potentially stronger or more recent selection signal at the *IRAK3* locus in this context. However, the permutation test, which involved shuffling the combined data for both loci 10,000 times, was not significant (*P* > 0.05).

*IRAK3* is a cytoplasmic homeostatic mediator of inflammatory responses and acts as a regulator of toll-like receptor (TLR) signalling pathways, which are crucial in innate immune responses and inflammation [33]. In humans, research has suggested that inflammatory processes influenced by *IRAK3* impact metabolic pathways in which the fat mass and obesity-associated gene (*FTO*) is involved, potentially linking the two genes in the context of metabolic syndrome and obesity-related inflammation [34]. Investigation by Claussnitzer et al. [34] indicated that inhibition of *IRAK3* in adipose tissue in mice reduced body weight and increased energy dissipation without a change in physical activity or appetite. Jia et al. [35] suggested that *IRAK3* may affect intramuscular fat in Yaks. *HMGA2* is a non-histone chromosomal protein that binds to AT-rich regions of DNA. It influences chromatin structure and gene expression by altering the architecture of DNA [36]. It has been associated with age at puberty and first rebreeding interval in tropically adapted beef cattle [37], precocity score and muscling in Nelore cattle [38], differences in size between dog breeds [39], and stature in cattle [4]. The protein encoded by *HMGA2* regulates the RNA-binding protein *IGF2BP2* (IGF2-binding protein 2), which in turn enhances translation of the *IGF2* gene [40]. *IGF2* binds to the IGF1 receptor (IGF1R) and the insulin receptor (IR), triggering signalling pathways that are critical for cellular growth and development. *IGF2* also can interact with components of the TLR signalling pathway, influencing the immune response. Given the current results and previous findings, we hypothesis that stress conditions can probably lead to increased DNA damage and thus a higher demand for DNA repair mechanisms, leading to co-expression of *HELB* and *IRAK3*. Considering the close link between DNA repair mechanisms and chromatin accessibility [41], efficient DNA repair likely necessitates accessible chromatin. Therefore, proteins such as HMGA2, which potentially modulate chromatin structure, may influence the efficiency of repair processes involving *HELB*. Our results cannot distinguish *IRAK3* or *HMGA2* as the causative gene in this QTL region (another possibility is both genes or their regulatory regions harbour a mutation affecting the traits).

We identified a region in the M-GWAS comprising of 5 SNPs spanning 37.1 to 37.9 Mb on Chr 6, with six genes in this region including *LAP3*, *LCORL*, *PPM1K*, *ABCG2*, *MED28,* and *FAM184B.* All variants had antagonistic effects on height/weight and heifer puberty/BCS, reflecting the negative genetic correlation between the timing of puberty and height in heifers. Many studies have identified this region as associated with livestock production traits, with different studies selecting different genes as the most likely to harbour a causative mutation. For example *FAM184B* is a candidate gene associated with meat traits [42], body weight [43], body composition traits [5], and litter size in sheep [44]. Other studies [45, 46] have reported several candidate genes affecting bone weight, such as *FAM184B, LAP3*, *MED28*, *LCORL*, and *ABCG2*. *LAP3*, as a member of the LAPs family involved in cell maintenance and growth development, had been suggested to be associated with body weight in sheep [47], body measurements traits in cattle [48]. *MED28*, a gene involved in the regulation of cell proliferation and cycle [49], has been reported as associated with body weight, intramuscular fat content, and yearling weight in cattle [50]. *LCORL* was also associated with calving ease [51], carcass and meat quality traits, as well as stature [52]. The gene coding protein phosphatase Mg^2+^/Mn^2+^-dependent 1K (*PPM1K*) and *ABCG2* (a member of the ATP-binding cassette family) have been previously reported as associated with milk fat and milk protein yield in cattle [53]. In human and mouse, *PPM1K* suppression has been reported to disturb energy metabolism homeostasis in the follicular microenvironment, providing an underlying mechanism of abnormal follicle development, thereby resulting in reproductive malfunction and metabolic disorders [54]. The lack of agreement between these studies makes it challenging to single out a single gene, however based on the EHH profile in this region, it seems that the SNP located at 37,266,497bp, could be the potential candidate affecting multiple traits, with evidence for strong recent selection at this locus. This SNP is located within the gene *FAM184B.*

The most significant variant on Chr 14 was located in intron 3 of *PLAG1* gene (23,338,890 T > G, rs109815800, P-value < 8.78 × 10^−44^), one of eight putative causative mutations previously identified in or close to this gene [55]. *PLAG1* initiates transcription of *IGF2*, a mitogenic hormone important for fetal growth and development, and has been implicated in genetic variation of stature in humans as well as cattle [4], and age of puberty in cattle [56]. This variant negatively affects gene expression of *UBXN2B* [9], a protein-coding gene involved in endoplasmic reticulum biogenesis [57], which was found to be associated with mid-test metabolic weight in Simmental x Angus crossbred [58], carcass weight, carcass fat, and carcass conformation in Simmental cattle [59], and carcass and growth traits in beef cattle [60]. The variant identified through integrating eQTL and M-GWAS data (Chr14:23,897,198), has strong positive correlation with the *PLAG1* SNP and positively and negatively affects the gene expression of *UBXN2B* and *BPNT2*, respectively. Previous work has shown that *BPNT2*-knockout mice exhibit impairments in total-body chondroitin-4-sulfation, leading to abnormal skeletal development (chondrodysplasia) [61]. The comparison between the AUC for EHH decay at two loci strongly suggested strong recent selection at the *PLAG1* locus, rather than *BPNT2* (Fig. 5). In line with current study previous study indicated that the introgressed *Bos taurus* allele at *PLAG1* region in Brahman animals increases stature, and the high frequency of the allele likely reflects strong selection for the trait [62].

In our analysis, six lead QTLs were identified as lead cis eQTLs, located within 2 Mb regions centered around the top association peaks detected through the M-GWAS analysis. These included one QTL on Chr 5, four on Chr 6—uncovered through iterative M-GWAS recalculations and conditional analyses—and one on Chr 14. While the top SNPs in the major regions on Chr 5, 6, and 14 did not directly overlap with any eQTLs, they exhibited the most significant associations. Therefore, we also reported and investigated three additional QTLs corresponding to these top signals for further functional evaluation.

Although there is a positive overall SNP effect correlation between the traits under study, ranging from 0.01 (BCS and height) to 0.54 (weight and height), the correlation between SNP effect estimates across traits for the nine potential eQTL/QTL candidates discovered in this study ranged from -0.90 (BCS and weight) to 0.98 (weight and height). Even at this stage of development (early reproductive age, around 600 days), it appears there is some competition for resources for growth and reproductive development, mediated by these loci. These findings underscore the complex genetic underpinnings associated with each trait and emphasize the importance of understanding the specific genomic regions influencing phenotypic variability in livestock populations. Identifying regions where selection could improve fertility without negatively affecting other traits offers new opportunities for selection. Among significant SNPs (P < 1 × 10^–9^) from the M-GWAS, we screened potential regions that had positive effects on all traits and identified regions on Chr 11 (53 Mb), 21 (1.9–2 Mb), and 30 (102 Mb and 114 Mb). These regions span a few genes, including *CTNNA2*, *SNRPN*, and *SNURF*.

Our results provide significant insights into the interplay between selection for height and its potential impact on other traits, such as fertility. Most cattle breeds have been subject to strong recent selection for height. Although we attempted to use EHH to assist in the identification of causative mutations, what our analysis really revealed was compelling evidence that selective sweeps for height-increasing alleles result in long regions of extended haplotype homozygosity. The localised extended linkage disequilibrium in these regions due to the selective sweeps is sufficiently large that it obscures identification of mutations that affect fertility and other traits with a pleiotropic association with height, complicating efforts to dissect the genetic basis of complex traits in cattle. Using high-parallel gene editing [63] as an optimal strategy for uncovering causative mutations could allow for the identification of these mutations by simultaneously targeting and modifying multiple genomic regions across many samples, paving the way for advancements in genetic research and the development of more effective breeding strategies.

## Conclusion

The four traits investigated in the current study are influenced by an intricate interplay of genes that regulate growth, metabolism, and reproductive development. Current results suggest that there is a correlation between the allele's influence on the timing of puberty in heifers (at six hundred days) and its impact on traits such as weight, height, and BCS. Specifically, the alleles on Chr 5, 6 and 14 that increase the likelihood of earlier puberty tend to be associated with lower weight and height but higher body condition score, and vice versa. The presence of longer EHH associated with height increasing alleles suggests that these loci are under strong positive selection. This selection, while beneficial for some traits, poses a challenge for the identification of causative mutations for fertility. These results give some indication of selection responses for multiple traits that are likely once these SNP are included on arrays used for genomic selection in beef cattle.

## Supplementary Information

Additional file 1: Table S1. The significant SNPs identified for various traits: Body Condition Score (BCS), height, weight, and heifer puberty. A total of 114, 6,405, 1,683, and 4,142 significant SNPs were found for each trait, respectively, with a significance threshold of P < 1 × 10–9. Table S2. Correlations calculated using signed t-values of candidate QTLs associated with corresponding gene expression and various phenotypic traits. T-values represent the ratio of the effect size to the standard error (a measure of variability or uncertainty in the estimate of the effect size) obtained from eQTL analysis or GWAS analysis.

Additional file 2: Figure S1. Manhattan plots and QQ plots of genome-wide association studies (GWAS) for four traits: a) Body Condition Score (BCS), b) Weight, c) Height, and d) Heifer Puberty. Each Manhattan plot displays the negative log10-transformed p-values of SNPs across the genome with the genome-wide significance threshold of P < 1 × 10^–9^. Figure S2. Manhattan plots depicting the Multi-trait GWAS analysis for height, weight, body condition score (BCS), and heifer puberty in 28,351 multibreed cattle, after filtering out variants with low imputation accuracy (Rsq < 0.4).

Additional file 3: Table S1. Most associated Single Nucleotide Polymorphism (SNP) per chromosome for each trait, as identified through single-trait GWAS analysis. Table S2. Most significant Single Nucleotide Polymorphism (SNP) positions per chromosome identified through multi-trait GWAS (M-GWAS) analysis (p-value < 1 × 10⁻⁹), along with the corresponding signed t-values (calculated as effect size divided by standard error) from single-trait GWAS analyses. Table S3. Significant Single Nucleotide Polymorphisms (SNPs) associated with gene expression (Forutan et al., 2024) and studied traits (i.e. Height, BCS, Weight, Heifer puberty), along with their potential regulatory effects on nearby genes. Table S4. The correlation between candidate quantitative trait loci (QTLs) across four traits including, body condition score (BCS), weight, height, and heifer puberty. The correlation were calculated based on SNP effect size obtained from single-trait GWAS analysis for each trait. Table S5. Effects of the candidate quantitative trait loci (QTLs) on four different traits as estimated from the original single-trait genome-wide association study (GWAS). Table S6. Pearson Correlation between each pair of traits calculated over the effects of nine candidate quantitative trait loci (QTLs) located on chromosomes 5, 6, and 14, estimated using single-trait GWAS analysis.

## Data Availability

Not applicable.
