# Supplementary material for: Selective sweeps for mutations increasing height impede identification of causative mutations for fertility and other correlated traits in cattle
Source: Genet Sel Evol. 2025 Oct 7;57:56. doi: 10.1186/s12711-025-01004-x (PMC12506307; doi:10.1186/s12711-025-01004-x)
Supplement: Supplementary file 2 — Additional file 2: Figure S1. Manhattan plots and QQ plots of genome-wide association studies (GWAS) for four traits: a) Body Condition Score (BCS), b) Weight, c) Height, and d) Heifer Puberty. Each Manhattan plot displays the negative log10-transformed p-values of SNPs across the genome with the genome-wide significance threshold of P < 1 × 10^–9^. Figure S2. Manhattan plots depicting the Multi-trait GWAS analysis for height, weight, body condition score (BCS), and heifer puberty in 28,351 multibreed cattle, after filtering out variants with low imputation accuracy (Rsq < 0.4). [file 12711_2025_1004_MOESM2_ESM.docx]

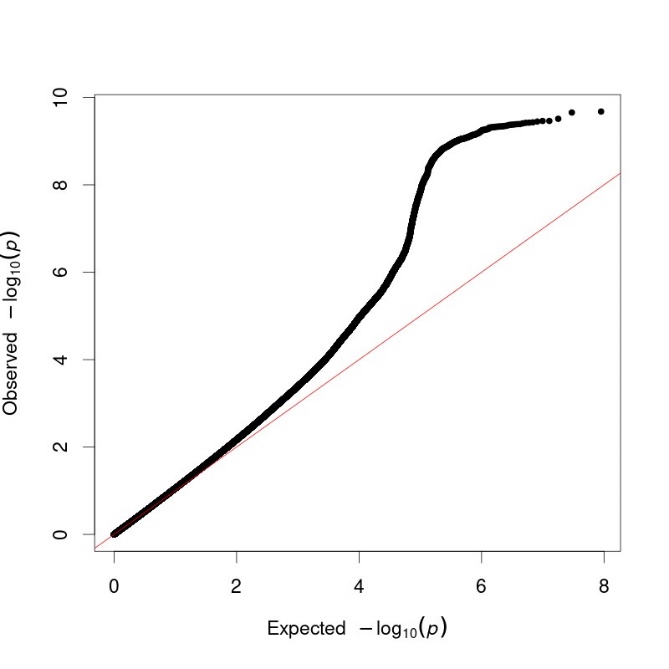

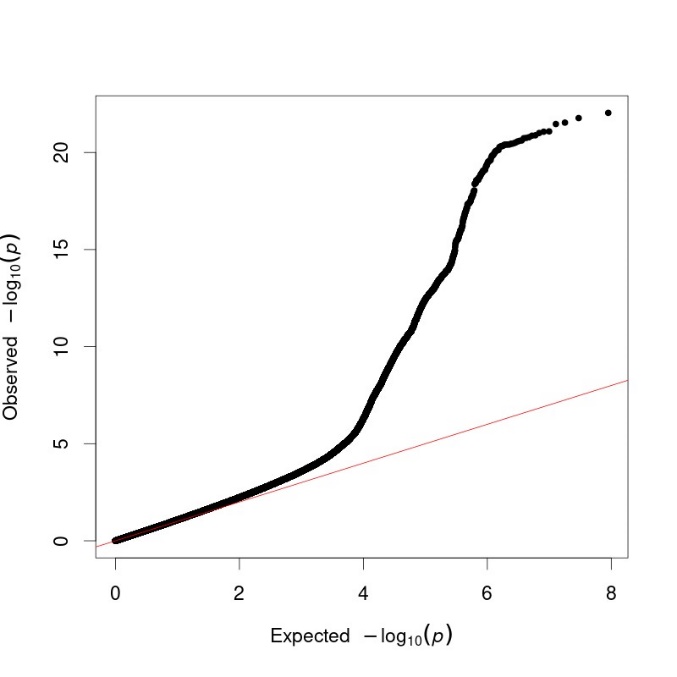

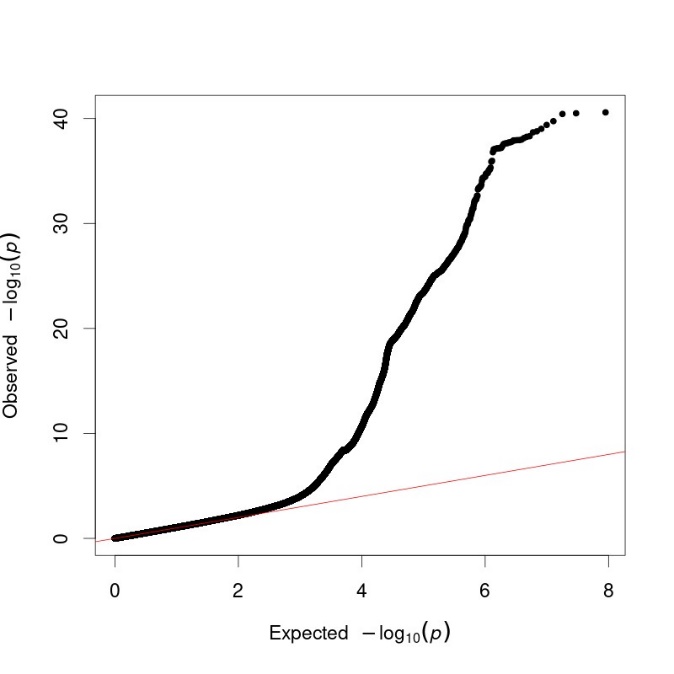

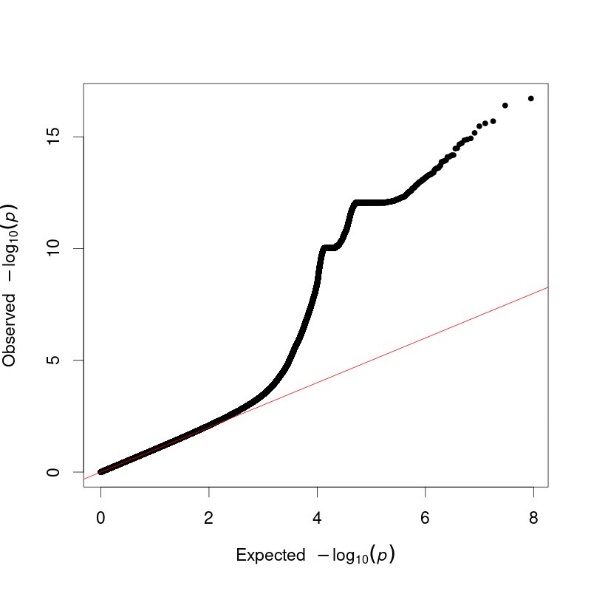

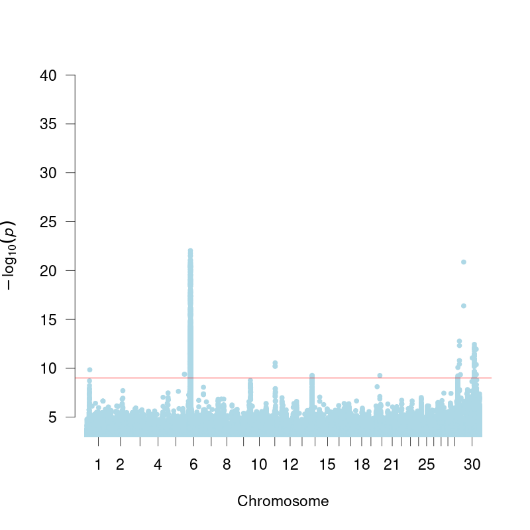

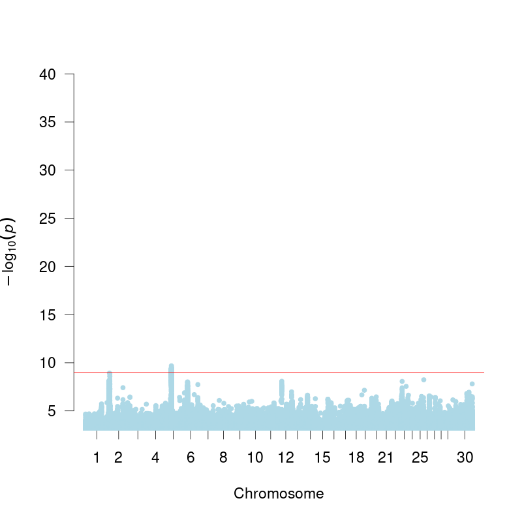

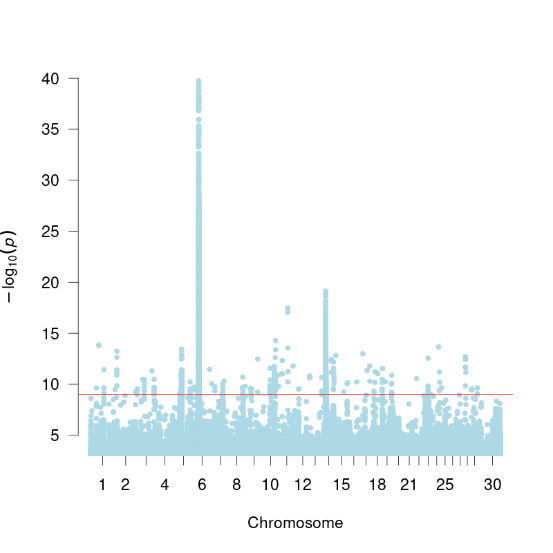

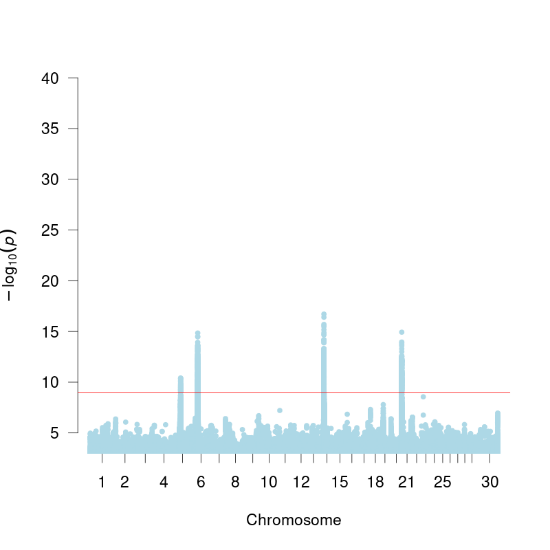


**a**

**b**

**c**

**d**

**Figure 1.** Manhattan plots and QQ plots of genome-wide association studies (GWAS) for four traits: a) Body Condition Score (BCS), b) Weight, c) Height, and d) Heifer Puberty. Each Manhattan plot displays the negative log10-transformed p-values of SNPs across the genome with the genome-wide significance threshold of P < 1×10^-9^


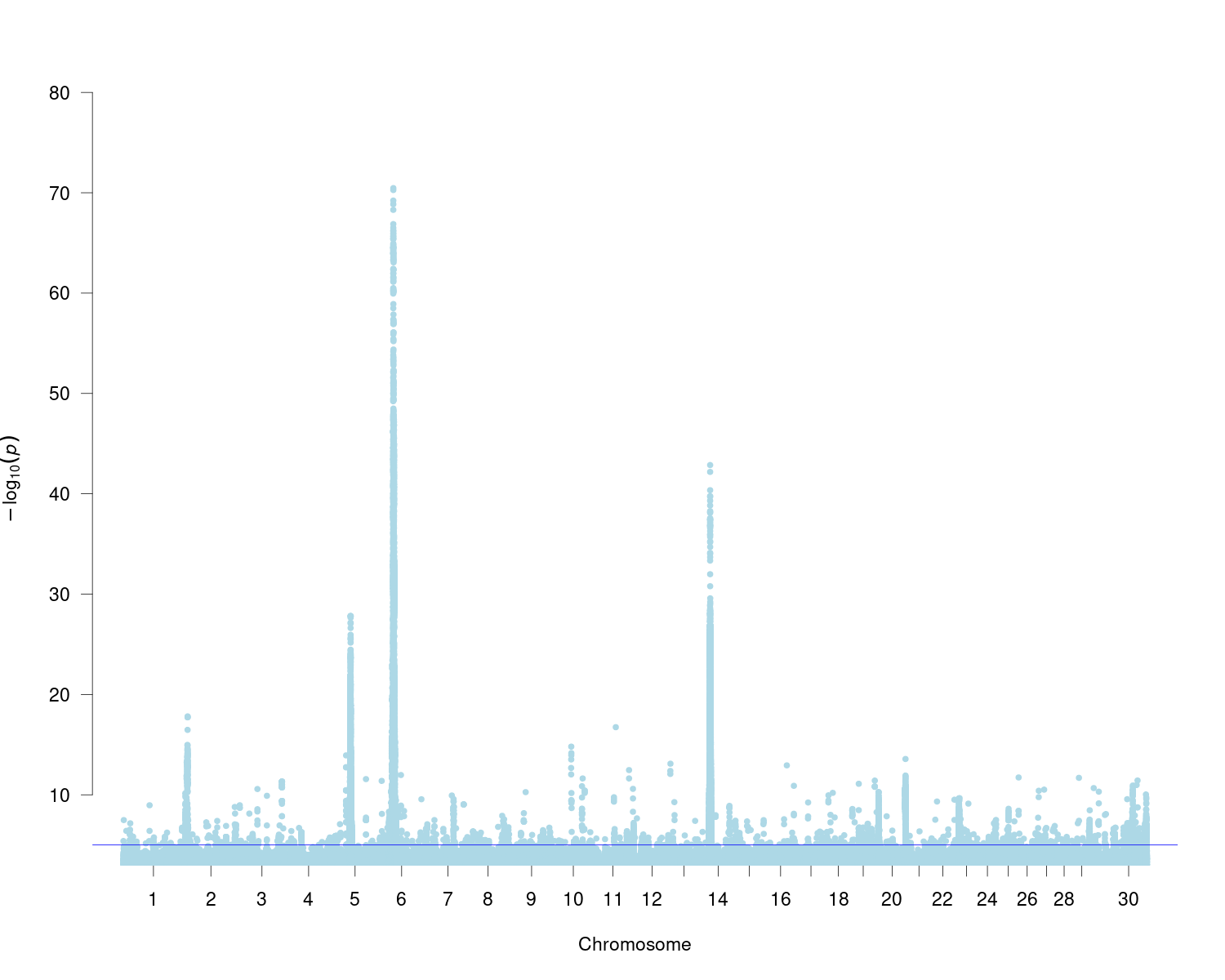


**Figure 2.** Manhattan plot depicting the Multi-trait GWAS analysis for height, weight, body condition score (BCS), and heifer puberty in 28,351 multibreed cattle, after filtering out variants with low imputation accuracy (Rsq < 0.4).
